# RNAtranslator: Modeling protein-conditional RNA design as sequence-to-sequence natural language translation

**DOI:** 10.1371/journal.pcbi.1013541

**Published:** 2025-10-03

**Authors:** Sobhan Shukueian Tabrizi, Sina Barazandeh, Helyasadat Hashemi Aghdam, A. Ercument Cicek

**Affiliations:** 1 Department of Computer Engineering, Bilkent University, Ankara, Türkiye; 2 Computational Biology Department, Carnegie Mellon University, Pittsburgh, Pennsylvania, United States of America; Chinese Academy of Science, CHINA

## Abstract

Protein-RNA interactions are essential in gene regulation, splicing, RNA stability, and translation, making RNA a promising therapeutic agent for targeting proteins, including those considered undruggable. However, designing RNA sequences that selectively bind to proteins remains a significant challenge due to the vast sequence space and limitations of current experimental and computational methods. Traditional approaches rely on in vitro selection techniques or computational models that require post-generation optimization, restricting their applicability to well-characterized proteins.

We introduce RNAtranslator, a generative language model that formulates protein-conditional RNA design as a sequence-to-sequence natural language translation problem for the first time. By learning a joint representation of RNA and protein interactions from large-scale datasets, RNAtranslator directly generates binding RNA sequences for any given protein target without the need for additional optimization. Our results demonstrate that RNAtranslator produces RNA sequences with natural-like properties, high novelty, and enhanced binding affinity compared to existing methods. This approach enables efficient RNA design for a wide range of proteins and even proteins with no RNA-interaction data available, paving the way for new RNA-based therapeutics and synthetic biology applications

## Author summary

RNA molecules play a vital role in controlling how cells function. Because of their ability to interact with proteins, RNAs are increasingly being explored as therapeutic tools—especially to target proteins that are difficult to treat with traditional drugs. However, designing RNA sequences that can bind to specific proteins is challenging, due to the massive number of possible sequences and the limitations of current laboratory and computer-based methods.

In this work, we present a new machine learning approach called RNAtranslator, which treats the design of protein-binding RNAs like translating one language (proteins) into another (RNAs). Using large datasets of known RNA-protein interactions, our model learns how these molecules communicate and can generate RNA sequences that are likely to bind to any given protein—including those never seen before. Unlike existing methods, our approach doesn’t require additional fine-tuning after generation and still achieves high novelty and strong binding potential. This opens the door to designing RNA-based therapeutics more efficiently and for a wider range of diseases.

## Introduction

Protein-RNA binding is an important component of the interactome and plays crucial roles in gene expression regulation [1], splicing [2], RNA stability [3], molecular degradation [4], RNA transport [5], and translation [6]. RNAs bind to proteins through either (i) RNA-binding domains (RBDs), which have rigid and well-defined 3D structures and selectively bind to specific RNA motifs, or (ii) Intrinsically Disordered Regions (IDRs), which have highly flexible structures, allowing them to interact dynamically with multiple binding RNA partners. The latter provides exciting opportunities for the design of novel RNA molecules that target and bind with proteins [7]. These can be used to inhibit, enhance, or modify RNA-protein interactions to restore functionality or eliminate disease-causing factors. RNA is a new and promising therapeutic agent to target proteins because only 15% of human proteins have binding pockets for ligands (small molecules) which are used as the common drug mechanism today [8]. This means that RNA has the potential to target proteins that have previously been deemed undruggable either with direct binding or by inhibiting the precursor RNAs. RNA-based drugs are also very favorable because (i) they have a lower immune system activation and inflammatory response with nucleoside-modifications; (ii) they have no genotoxicity risk as they do not integrate into the genome; (iii) they are relatively swift and inexpensive to produce with optimized chemistry and delivery [9].

The bottleneck in protein-conditional RNA design is the sheer number of possibilities for the candidate sequence, and unfortunately, current practice in the laboratory relies on manual and experimental design. The first strategy is to generate a large pool of candidate binding RNA sequences for a target using tools such as Primer-BLAST [10] followed by in vitro filtering of the library using experimental validation approaches such as SELEX (Systematic Evolution of Ligands by EXponential Enrichment) [11] which becomes more stringent in every validation cycle. The key element for success when it comes to selecting a good library to start with is based on luck, which is clearly not desirable. The second approach begins with an existing solution to a related problem, if one can be found, which is then optimized through the same in vitro test cycle. Both methods cost significant time and money and produce a suboptimal design. Computational techniques can aid design but due to their key limitations they are limited to proof-of-concept.

Most computational RNA design methods focus on *unconditional* RNA design. While early efforts solve the inverse folding problem via optimization [12–23], recent works use deep/reinforcement learning to design RNA structure (2D or 3D) [24–30] or RNA sequence [31–35]. Although these methods can design synthetic RNA with natural-like properties such as GC content and minimum-free energy, only a few are capable of designing RNAs to target specific proteins (i.e., protein-conditional design). RNAFlow [27] is the only model that can design a *structure* to target a protein which uses a conditional flow-matching method. However, state-of-the-art structure designing models including RNAFlow cannot design novel molecules with variable length and the models can only recapitulate structures in the dataset [29]. This is because RNA is structurally very flexible compared to proteins and the paucity of ground truth structures bar training deep learning models [36]. Thus, the performances are random when designing conformationally diverse RNAs [27].

Protein-conditional RNA *sequence* design models do not suffer from training data scarcity and learn a latent distribution over widely available RNA sequence databases using architectures like generative adversarial networks [31,33] and GPT-based language models [34]. However, conditioning on the target requires a post-generation optimization step with a binding affinity prediction method for the target protein. This means the current state-of-the-art procedure has key bottlenecks: (1) The two-step approach (generation+optimization) does not harness the full potential of learning better embeddings for RNA in relation to other molecules (targets); (2) The pipeline is dependent on the availability of RNA interaction data for the target and/or third-party tools that predict the binding affinity of the designed RNA with the target protein. For example, such DeepBind [14] models are available for only 732 proteins and DeepCLIP models [37] are available for 221 proteins. This means it is not possible to design RNA to target under-studied proteins or novel synthetic proteins. This is a crucial bottleneck for the applicability of this system as the number of proteins in the human body is estimated to be between 20,000 to several millions depending on the definition [38–40].

In this study, we propose a novel approach to overcome the challenges mentioned above. This first-of-its-kind method enables the design of binding RNA for *any* target protein. That is, for the first time, we formulate the problem of protein-conditional RNA design as a sequence-to-sequence *natural language translation* problem (not to be confused with biological process of protein generation). We use large-scale RNA-protein interaction datasets to train a generative language model, RNAtranslator, end-to-end. Like ChatGPT inputing a sentence in English and translating it into French, RNAtranslator inputs a target protein sequence and *translates* it to a novel binding RNA sequence. The model learns a latent space for both the RNA and the protein along with their interactions, and thus does not require any post-processing or optimization method to tailor the design for binding to the target. Thus, RNAtranslator can design a binding RNA for a target protein with no information other than its sequence for the first time. While this does not mean all proteins are targetable, our approach opens the doors to study understudied, novel or synthetic proteins with little or no interaction information available.

We design binder RNAs for 11 randomly selected proteins and 3 therapeutic target proteins and assess the preformance using (i) binding affinity estimations, (ii) docking simulations, and (iii) molecular dynamics analyses. We show that it is capable of generating novel RNA sequences that closely resemble targets’ natural binders in terms of binding affinity, dynamics, GC content, minimum free energy (MFE), and ensemble free energy distributions. We further test the model on more challenging scenarios, including a protein with no known RNA interactiond (i.e., PRP4K) and a protein which was not seen during training (i.e., PRPF8). The model still designs novel RNAs with strong binding affinity in these settings, showing its ability to learn generalizable features of RNA–protein interactions. These capabilities make RNAtranslator the first model that can virtually design RNAs for new targets with only sequence information. Our model a key step toward programmable RNA-based therapeutics. The model and code are available at github.com/ciceklab/RNAtranslator.

## Results

### RNAtranslator overview

**Fig 1:**
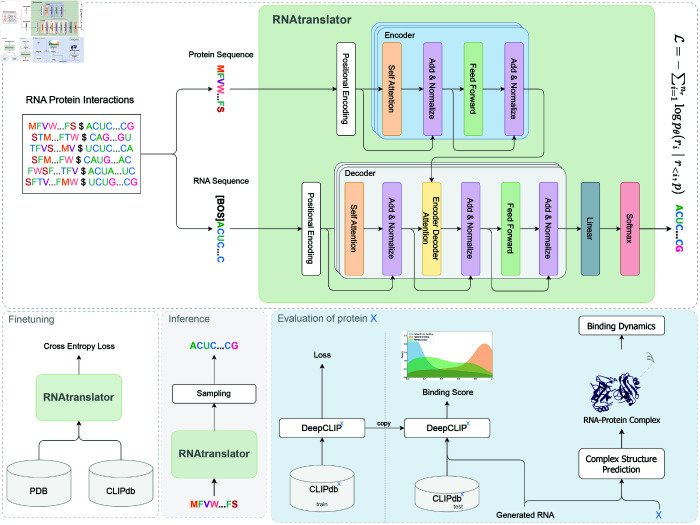
Overview of RNAtranslator pipeline The model follows an encoder-decoder transformer framework, where the encoder processes the protein sequence using positional encoding and encoder blocks to extract an embedding. The decoder takes the RNA sequence input, applying self-attention, encoder-decoder attention, and feed-forward layers to learn to generate target-specific RNA sequences. Training occurs in two steps: Large scale pretraining with experimental and computationally predicted interactions followed by fine-tuning with experimental interactions. During inference, the model requires only the protein sequence as input and generates novel RNA sequences through iterative sampling. We evaluate the designed RNAs in two ways: (i) we predict their in-silico binding affinity to protein X with DeepCLIP^*X*^, a model trained on the CLIPdb^*X*^ subset that contains only interactions for protein X and is pre-split into training and test sets; and (ii) we analyse each RNA–protein complex by molecular-dynamics simulation.

RNAtranslator is an encoder-decoder transformer-based language model designed for RNA sequence generation conditioned on a target protein sequence input. The model aims to generate RNA sequences that exhibit strong binding affinity to target protein while maintaining structural stability and natural RNA-like properties. The architecture of RNAtranslator is shown in Fig 1. We trained the model in two steps: (i) we first trained it using 26 million RNA-protein interactions from the RNAInter dataset, which includes both computational and experimental data, and (ii) we then fine-tuned it with 12 million experimentally validated interactions. The model encoder is input with the target protein sequence. The decoder inputs the binding RNA sequence and the embedded protein sequence and learns to regenerate the RNA sequence from left to right. During inference, the model inputs the target protein sequence and generates novel RNA sequences that are likely to bind to it. We select the candidate sequence after sampling from this step.

To evaluate our designs, we use in silico binding affinity predictors such as DeepCLIP [37] and docking tools to simulate the molecular interaction between the design and the target structure. We also use OpenMM [41] to simulate the dynamics of our design bound to the target protein. Molecular dynamics simulations help reveal how these complexes behave in a physiologically relevant environment rather than a static crystal structure. These simulations provide insights into conformational changes, hydrogen-bonding networks, and the thermodynamics of binding at atomic resolution by using force fields optimized for both proteins and RNA. Please see section Methods for the details on the simulation.

In the upcoming subsections, we first evaluate the RNAtranslator by generating binding RNAs for three therapeutically important proteins: p53, thrombin, and EGFR. Then, we evaluate the novelty of the RNA sequences generated using RNAtranslator. This step ensures that our generated RNAs are novel compared to existing known RNA sequences that bind with the same targets. Subsequently, we design RNAs to bind with RNA-binding proteins RBM5 and ELAVL1 and compare their binding affinities with (i) RNA sequences generated by state-of-the-art methods, (ii) natural known binding RNAs of these proteins, and (iii) randomly selected natural RNAs that are unlikely to bind. To further test the generalizability of RNAtranslator, we analyze its ability to design binding RNAs for unseen proteins. The PRPF8 and PRP4K interactions were not included in our training data. By predicting and validating RNA–protein interactions through docking and molecular dynamics simulations, we confirm that our approach can effectively target both novel and understudied proteins. We also extend our analysis to 9 more RNA-binding proteins to determine the broader applicability of our RNA design method. Finally, we analyze the stability of the generated RNAs to confirm their structural robustness and thermodynamic favorability.

### RNAtranslator designed RNAs which are predicted to bind to therapeutically important proteins

Designing RNA molecules that bind to therapeutically important proteins enables targeted therapy. In this subsection, we focus on three distinct protein targets which are therapeutically important: p53, thrombin, and the epidermal growth factor receptor (EGFR). These proteins are biologically and structurally diverse. More specifically, they are (i) a nuclear transcription factor, (ii) a secreted serine protease, and (iii) membrane-bound receptor tyrosine kinase, respectively. Despite their diversity, each plays a critical role in disease progression, especially in cancer and cardiovascular disorders, and they are well-established drug targets. Note that, unlike typical RNA-binding proteins, EGFR is a membrane receptor tyrosine kinase that does not naturally bind RNA, making it a particularly challenging target for RNA design [42].

**Fig 2:**
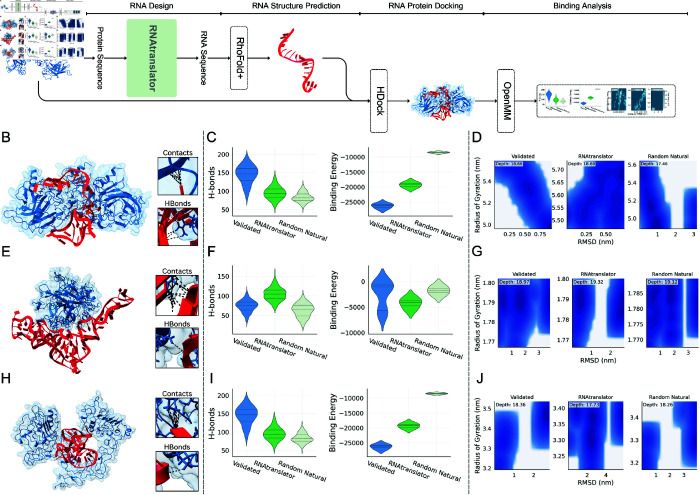
Evaluation of designed RNA molecules to bind to three therapeutic target proteins: p53, thrombin and EGFR (A) The figure shows the full pipeline for designing RNA sequences that bind to target proteins. Given the the target protein’s sequence, RNAtranslator generates an RNA sequence to bind it. The 3D structure of the designed RNA sequence is predicted using RhoFold+, and then the RNA-Protein complex is modeled using HDock. Finally, the simulations are conducted to test how well the RNA binds. (B–J) Evaluation of the designed RNA for three protein targets: p53 (Panels B–D), thrombin (Panels E–G), and EGFR (Panels H–J). For each target, the designed RNA is compared with two RNA sequences: a validated RNA known to bind the target, and a randomly selected natural RNA sequence. (B, E, H) Visualization of the three-dimensional structures of the RNA-protein complexes formed after molecular dynamics simulations using the designed RNA sequences. The zoomed-in panels highlight atomic contacts and hydrogen bonds at the interaction interface. (Panels C, F, I) Distributions of hydrogen bonds and binding energies are shown, these distributions are observed during molecular dynamics simulations. The designed RNA sequences form a similar or greater number of hydrogen bonds compared to validated aptamers, and more than random natural RNAs. Additionally, the binding energies of the designed RNAs are lower (indicating stronger binding) than those of random RNAs, and are often comparable to or better than those of the validated aptamers. (Panels D, G, J) Energy landscapes based on root-mean-square deviation (RMSD) and radius of gyration (RG) during molecular dynamics simulations. For p53 and thrombin (Panels D and G), the designed RNA sequences converge to stable conformations characterized by low RMSD and compact structures, comparable to those of the validated aptamers. In contrast, for EGFR (Panel J), the designed RNA does not exhibit a clearly stable structure, displaying more variability in both conformation and compactness.

The summary of our evaluation pipeline for designed RNA sequences is shown in Fig 2A. We start by obtaining the high-resolution 3D crystal structure of the target protein from the Protein Data Bank [43] (PDB IDs: 1TUP [44,45] for p53, 4DII [46,47] for thrombin, and 1NQL [48,49] for EGFR). Native ligands or aptamers are removed from the structure, if any. If the ground truth structure is not available, one can use AlphaFold3 to predict the structure [50]. Only the target protein sequence is used as input to RNAtranslator to design binding RNA sequence. Next, we predict the structure of the designed RNA using RhoFold+ [51]. We then model the RNA-protein complex using HDOCKlite [52]. Finally, we run molecular dynamics simulations with OpenMM [53] to analyze dynamics of the complex. The details of MD simulation procedure is mentioned in Sect Methods. For these proteins, we compare our designed RNA with two groups of RNAs (i) experimentally validated aptamers, meaning RNA that designed to bind these proteins and whose high affinity is confirmed by experimental methods [54–57], and (ii) randomly chosen natural RNA as negative controls.

We visualize the RNA–protein complex after the simulation to see whether the partners remain bound. The results for each target is shown in Fig 2B, 2E, and 2H for p53, thrombin, and EGFR, respectively. The snapshots next to the visualizations in Fig 2 show that the interfaces contain contacts and hydrogen bonds. To follow the interaction more closely, we analyze three time-resolved metrics for every complex: (i) The number of hydrogen bonds, (ii) The binding energy, and (iii) The free-energy landscape (FEL) as shown in Fig 2. We describe how we calculate each of these metrics in Sect Methods. Note that the distributions for the number of hydrogen bonds and the binding energies are for a single RNA-protein complex throughout the simulation procedure.

For all targets, our designs make similar or more H-bonds compared to validated RNAs and more H-bonds than random RNAs (Fig 2C, 2F, 2I). For thrombin (Fig 2F), RNAtranslator outperforms both baselines, achieving the highest hydrogen bond count. Similarly, in terms of binding energy and stability, RNAtranslator outperforms random RNA for all proteins and closely matches the performance of validated aptamer.

The Free Energy Landscape (FEL) maps show the structural changes of the complex by using the first simulation frame as a reference and calculating, for each frame, the root-mean-square deviation (RMSD) to measure atomic displacement and the radius of gyration (RG) to assess compactness. As shown in the FEL maps (Fig 2D, 2G and 2J), for p53, our RNAtranslator RNA shares the same deep basin as the validated aptamer, both appearing at low RMSD and a compact RG, while the random RNA sits in a shallower pocket with larger structural drift. In thrombin, the basin carved out by our design is the deepest of all and coincides with the smallest RMSD values, indicating the most rigid binding mode. In contrast, for EGFR the basin formed by our RNA is shallower, appears at a higher RMSD, and spreads to a larger RG than others; it even trails the random natural RNA. This suggests a lower structural stability of the complex; however, strong binding can still occur, as indicated by a high number of hydrogen bonds and low binding energy, which reflect favorable interactions between RNA and the target.

We also compare the secondary structures of the validated aptamers and our designs (which is predicted by RhoFold+) in S2 Fig. In every case, our designs show the expected mix of long, base-paired stems that stabilize the scaffold and loop or bulge regions that remain free to contact the protein. We mark the nucleotides that contact with the protein after the MD simulation and observe that they cluster in—or near to—those unpaired segments, confirming that accessible loops are essential for binding [58]. To compare thermodynamic stability, we compute the length-normalized minimum free energy (MFE) of each RNA. For EGFR (panel A) our design, reaches virtually the same normalized MFE as the validated aptamer while packing in more hairpin loops and a slightly higher GC fraction. These are features that may further stabilize local structure [59]. For p53 the validated RNA is more stable, yet our sequence still forms the key stems and loops required for binding (see S2B Fig). For thrombin, our design folds into a lower (more favorable) MFE and adopts a richer stem and junction pattern than validated RNA (see S2C Fig).

### RNAtranslator designs novel RNAs with high binding potential

We perform a *novelty* analysis to check whether the model generates novel sequences or simply regenerates natural RNAs from the training dataset. To test this, we compare the designed RNA sequences with naturally occurring binding RNAs using BLAST (Basic Local Alignment Search Tool). To make it more accurate, we first separate the RNA sequences into short and long groups based on a 50-nucleotide threshold and adjust the BLAST word size—using 4 for short sequences and 7 for long ones. Next, we compare each sequence with the target proteins’ binding RNA database by applying the following thresholds: (i) 70% identity, (ii) a minimum alignment length of 15 base pairs, (iii) 50% query coverage, and (iv) an E-value of 0.1. Sequences that meet these criteria are classified as *similar to known*, while those that do not are considered novel.

Here, we compare RNAtranslator with only two other methods that can perform protein-conditional RNA design using the target sequence: RNAGEN [31] and GenerRNA [34]. RNAGEN is a generative adversarial neural network that generates RNA sequences *de novo*. In a post-processing step seed RNAs are then optimized to bind to the target using a binding affinity predictor’s rewards. GenerRNA is a GPT-based generative language model which can design RNAs *de novo* as well. The model needs to be fine-tuned with the target protein’s RNA interactions for conditional design.

We generate 500 RNAs using each method and compare the percentage of novel RNA sequences in the designed sets using the criteria mentioned above. We select two proteins with substantially different numbers of known RNA interactions from the CLIPdb database: RBM5, which has a relatively small set of interactions (3,917 interactions), and ELAVL1, with a notably large set of interactions (1,079,145 interactions). This selection allows us to analyze how the size of the training dataset affects the novelty of generated RNA sequences. RBM5 protein is an RNA binding protein that plays a role in apoptosis through pre-mRNA splicing of target genes. Its role is well characterized in cancer and is a candidate drug target for other conditions such as osteoprosis [60] and central nervous system disorders [61]. ELAVL1 is also an RNA binding protein that stabilizes mRNAs by binding to AU-rich regions. It has also been associated with cancer [62]. Thus, successfully designing RNA to target these proteins holds therapeutical potential.

Our results show that 83.4% of the RNAtranslator-designed RNAs for RBM5 are novel. For ELAVL1 71.2% of the designed sequences are novel. GenerRNA yields a substantially lower novelty rate, suggesting the fine-tuning procedure for the target protein leading to overfitting. Only 56.76% and 56.4% of the designed sequences are novel for RBM5 and ELAVL1, respectively. RNAGEN achieves a comparable novelty rate for RBM5 (81.64%) and higher novelty rate for ELAVL1 (85.94%). Note that RNAGEN does not directly use interaction data but uses a third-party affinity predictor tool to customize the designed RNA which makes it less likely to produce known RNAs.

**Fig 3:**
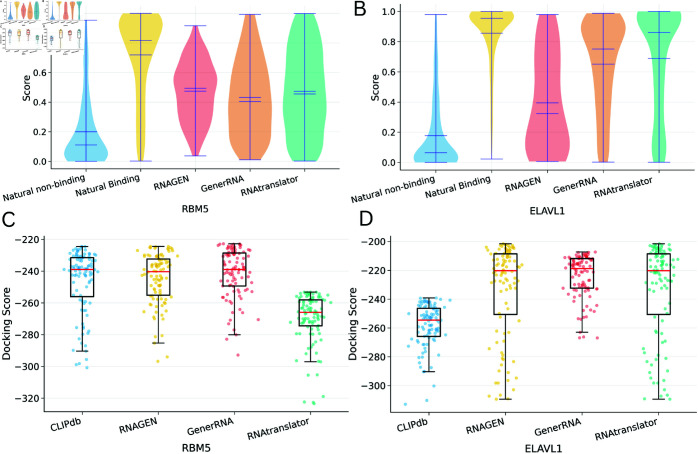
Comparison of predicted binding affinities between generated and natural RNAs for RBM5 and ELAVL1 proteins as targets (A) Distribution of binding affinities for RBM5 protein predicted by DeepCLIP. (B) Distribution of binding affinities for ELAVL1 protein predicted by DeepCLIP. (C) HDOCKlite scores for the top 100 models for RBM5-RNA complexes. (D) HDOCKlite scores for the top 100 models of ELAVL1-RNA complexes. For HDOCKlite scores, lower values indicate better docking (stronger predicted binding).

We filtered out known RNAs from all groups and compared the binding affinities of novel RNA sequences to natural RNAs using DeepCLIP. For each protein target, we selected the RNA sequence with the highest binding affinity score and performed molecular docking analysis using HDOCKlite, which provided the top 100 docking models indicating potential binding positions. In Fig 3, panels (A) and (B) show the distributions of binding affinities for RBM5 and ELAVL1 proteins, respectively. Overall, all methods performed better for ELAVL1, likely due to significantly larger training data. Additionally, RNAs generated by RNAtranslator generally showed higher binding affinity than RNAs produced by other methods. Panels (C) and (D) show docking score distributions for the top 100 docking models of RBM5 and ELAVL1, respectively, where lower scores indicate better binding. For RBM5, while other tools’ docking score distributions resemble natural binders’ distribution, RNAtranslator-generated RNAs achieve a distribution whose mean is lower than even experimentally validated natural RNAs’ distribution. This highlights the effectiveness of our model in designieng high-binding-affinity-RNAs with a relatively small set of RNA interactions. For ELAVL1, our model achieves the closest mean binding affinity to the natural binders of the protein.

**Fig 4:**
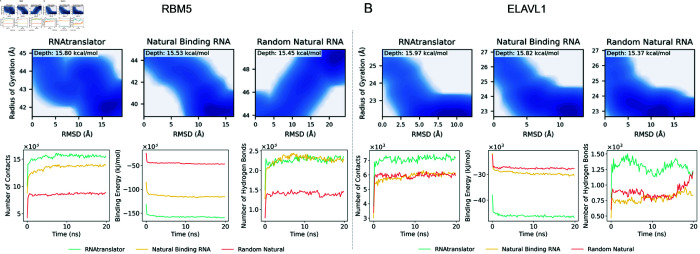
Molecular dynamics simulations showing interactions of RBM5 and ELAVL1 with RNAs generated by RNAtranslator and Natural RNAs (A) and (B) show simulation results for two RNA-binding proteins: RBM5 and ELAVL1, respectively. For each protein, three RNA sequences are compared: the RNA designed by RNAtranslator, a natural RNA known to bind the protein, and a randomly selected natural RNA. The top row displays free energy landscapes projected onto root-mean-square deviation (RMSD) and radius of gyration (RG). RNAtranslator achieves energy minima that are comparable to or deeper than those of natural binding RNAs. The bottom row presents simulation metrics over a 20-nanosecond trajectory. RNAtranslator forms more atomic contacts and achieves lower (i.e., more favorable) binding energies than random RNAs, and performs on par with or better than the natural binders. Hydrogen bonding distributions further support the binding of the predicted RNA-protein interactions.

We also evaluate how the proteins RBM5 and ELAVL1 interact with RNAs designed by RNAtranslator using molecular dynamics simulations (see section Methods). As shown in Fig 4, RNAs generated by RNAtranslator form more contacts and have lower (better) binding energies with both RBM5 and ELAVL1 compared to natural binding RNAs and random natural RNAs. FEL analysis also cornfirms this. For ELAVL1, our design reaches the maximum basin depth of 15.97*kcal*/*mol*, compared with 15.82*kcal*/*mol* for the natural binder and 15.37*kcal*/*mol* for the random control. Similarly for RBM5, these numbers are as follows: 15.80*kcal*/*mol*, 15.53*kcal*/*mol* and 15.45*kcal*/*mol*, respectively. Deeper basins confirm that RNAtranslator generates complexes that remain in a more stable, low-energy state throughout the simulation, aligning with the stronger binding-energy profiles and larger contact counts. Please see S1 Text for an analyses of the length distribution of the designed RNAs and robustness of the model to noise in the input protein sequences.

While we show that RNAtranslator designs novel RNAs with better predicted binding affinity, it would have been just another method with incremental improvements. The most important advantage and novelty of the our method over the others is that it does not require additional information for the target protein. This enables RNAtranslator to design RNAs to target understudied or novel/synthetic proteins. Note that to use RNAGEN and GenerRNA, we need post-processing of the model or the designed RNAs. That is, we need to optimize the RNAs generated by RNAGEN to have them bind to RBM5 and ELAVL1 using a specific protein-target specific binding affinity predictor trained. Similarly, we need to fine-tune the base GenerRNA model with RNAs that are known to bind to RBM5 and ELAVL1, respectively. These tools/data might not be available at all, prohibiting the conditional design. RNAtranslator is free from these steps, which enables it to design a binding RNA to a target protein using only its sequence. It does not require interaction data or third-party binding affinity estimators. We demonstrate this is possible in the next section with a protein that does not have a binding affinity predictor or known RNA interactions. Thus, RNAGEN and GenerRNA cannot design RNAs to target this protein.

### RNAtranslator can design for a protein without available RNA interaction data

To test how well our model generalizes, we evaluated it on two proteins not included in the training: PRPF8 and PRP4K. PRP4K is a kinase responsible for pre-mRNA splicing, signal transduction, and tumor suppression [63]. There are no known RNA interactions for this protein in RNA interaction databases which means it is not possible to design RNA to target PRP4K with current methods. PRPF8, on the other hand, is a core component of the U5 small nuclear ribonucleoprotein (snRNP) complex within the spliceosome. It is essential for the precise removal of introns during pre-mRNA splicing by orchestrating the interactions between pre-mRNAs and small nuclear RNAs (snRNAs) [64]. There are 27,046 interactions for this protein in the CLIPdb database, which we did not use during training but instead used to evaluate our model with the DeepCLIP method.

**Fig 5:**
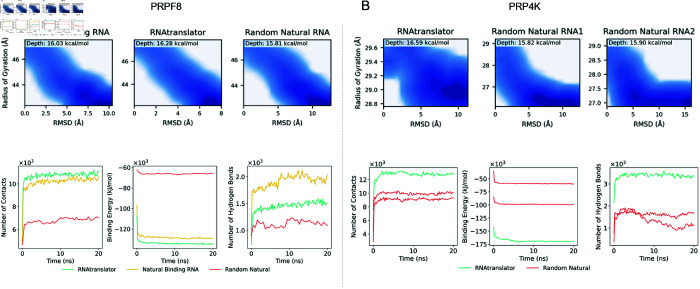
Evaluating RNAtranslator generalization to unseen proteins and robustness (A) For PRPF8, we compare designed RNAs, natural binders, and random RNAs using molecular dynamics. RNAtranslator sequences form more hydrogen bonds and show lower (better) binding energy than random RNAs. Their free-energy landscape (FEL) also shows a deeper basin than the natural and random RNA, indicating stable binding. (B) For PRP4K, RNAtranslator sequences again show more contacts, stronger binding energy, and more hydrogen bonds than random RNAs. FEL plots confirm that the RNAtranslator complex reaches the deepest energy basin, showing stable and strong binding.

We cannot predict the binding affinity of the designed RNA sequences for PRP4K using DeepCLIP as there is no interaction data to train such an affinity predictor. To evaluate the generated RNA for this protein, we get the ground-truth crystal structure of PRP4K available in the Protein Data Bank (4IAN [46,65]). Analysis of hydrogen-bond and contact plots in Fig 5B reveals that the RNAtranslator generated RNA bound PRP4K consistently forms more contacts compared to randomly selected natural RNAs. Throughout the simulation, the RNAtranslator-generated RNA maintains more hydrogen bonds relative to these other RNAs. This increased number of hydrogen bonds indicates stronger interactions, which is further confirmed by the binding energy analysis. The RNAtranslator generated RNA bound PRP4K demonstrates binding energies that are more negative than the natural RNAs, highlighting its better binding affinity and stability. Additionally, the free-energy landscape (FEL) analysis supports these findings. The Natural Binding RNA unbound protein shows a basin depth of approximately 15.82*kcal*/*mol*, whereas the RNAtranslator-generated-RNA-protein complex achieves a deeper basin of around 16.59*kcal*/*mol*.

PRPF8 is a large protein containing 2335 amino acids, to fit to our model we truncate the first 400 amino acids, which are not annotated as binding domains in UniProt, reducing the protein to 1935 amino acids. Using RNAtranslator, we generated 500 RNAs for PRPF8 and compared them with natural binding RNAs using the DeepCLIP model. S5 Fig shows that the distribution of binding scores for RNAtranslator RNAs closely matches that of natural binding RNAs. Additionally, for more analysis the structure of PRPF8 is predicted using AlphaFold3 [50] and through molecular dynamics simulations, we analyzed the interactions within the complexes. The results in Fig 5A show that, while the number of hydrogen bonds for RNAtranslator’s RNA is less than natural binding, the binding energies are lower and number of contacts are higher, confirming it’s stronger binding interactions compared to non-binding RNAs. FELs in the Fig 5A, also show the same. The RNAtranslator generated RNA achieves a deepest basin of 16.28*kcal*/*mol*, while the complex of natural binding RNA reaches 16.03*kcal*/*mol*.

### RNAtranslator can design RNAs with high predicted binding affinity to Nine targets

We evaluate the binding affinity of RNAs generated by RNAtranslator for nine proteins listed in CLIPdb. Specifically, we generate 128 RNAs each for ZC3H7B, AGO2, U2AF2, RBM5, HNRNPA1, TARDBP, MOV10, and SRSF1. By assessing these RNAs using the DeepCLIP model, we analyze the distribution of binding scores, allowing us to compare RNAtranslator-generated RNAs to natural binding and natural non-binding RNA sequences. These natural binding and non-binding RNAs are selected from an independent test set, ensuring that the DeepCLIP model has not been trained with these specific sequences, thus maintaining the integrity of our assessment.

**Fig 6:**
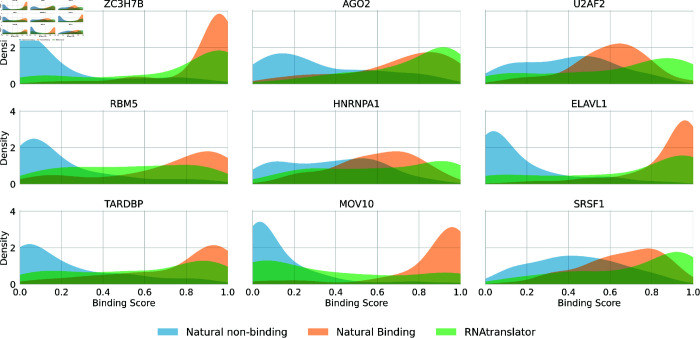
Binding affinity comparison of RNAtranslator-generated RNAs and natural RNAs RNAtranslator-generated RNAs show high binding affinities across nine protein targets. The distributions of binding scores indicate that RNAtranslator-generated RNAs generally show affinities comparable to naturally binding RNAs, with substantial overlaps observed for most proteins.

As shown in Fig 6, RNAtranslator-generated RNAs generally show binding affinities comparable to naturally binding RNAs for most targets, notably for proteins such as AGO2, and SRSF1, where distributions overlap substantially. This overlap indicates that the sequences produced by RNAtranslator have a binding profile similar to naturally occurring RNAs, reflecting the model’s accuracy in capturing essential characteristics necessary for effective protein binding. Furthermore, a substantial difference between the distributions of natural binding and non-binding RNAs supports the robustness of the DeepCLIP predictions, confirming its effectiveness in distinguishing functionally relevant RNA-protein interactions. Together, these observations emphasize RNAtranslator’s capability to produce sequences with meaningful binding affinities across a range of protein targets, underscoring its utility for advancing research in RNA biology and developing RNA-based therapeutic strategies.

### RNAtranslator generates RNAs that are predicted to be stable

To evaluate the stability of RNAtranslator-generated RNA sequences, we analyzed key stability metrics, including free energy, GC content, and ensemble free energy unfolding. The Minimum Free Energy (MFE) serves as an indicator of RNA stability by predicting the most thermodynamically favorable secondary structure. Lower MFE values suggest more stable conformations, as they represent the minimum energy required for an RNA molecule to fold [66]. Furthermore, GC content was examined as a complementary measure, since a higher GC content enhances structural robustness through stronger base-pairing and higher melting temperatures [67].

**Fig 7:**
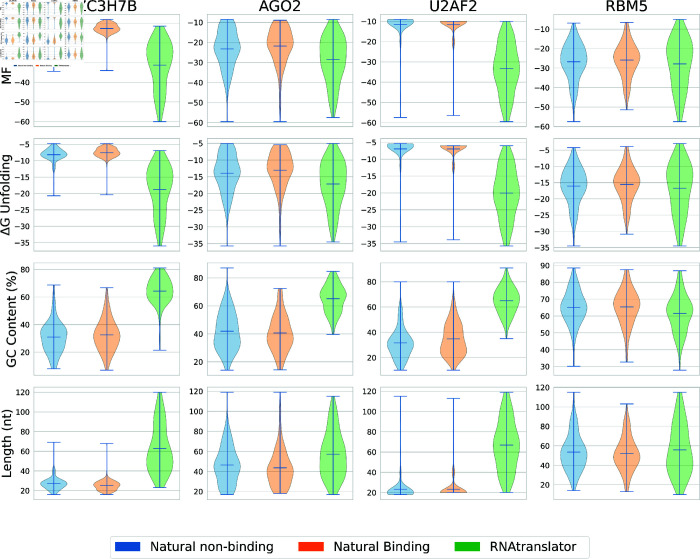
Stability analysis of RNAtranslator generated RNAs compared to natural RNAs. Minimum Free Energy (MFE) distributions is shown in the first row, where RNAtranslator-generated RNAs achieve stability levels comparable to natural binding RNAs, reinforcing their thermodynamic favorability. Ensemble Free Energy ($\Delta G_{ensemble}$) distributions, further validating the structural stability of RNAtranslator-generated RNAs in a thermodynamic ensemble. GC content comparison, indicating that RNAtranslator-generated RNAs closely match the natural binding RNAs, suggesting structural robustness. Distribution of RNA sequence lengths across different groups, showing that RNAtranslator-generated RNAs exhibit a broad yet biologically relevant length distribution.

Our results show that RNAtranslator-generated RNAs are stable with respect to these metrics. The analysis of GC content in Fig 7 shows that natural binding RNAs tend to have a similar GC content compared to nonbinding natural RNAs, as expected. RNAs designed by RNAtranslator have even a stronger GC composition compared to natural binding RNAs in four columns. Our analysis on MFE also further supports these findings. As shown in Fig 7, RNAtranslator generated RNAs achieve a distribution slightly more stable than natural RNAs. This suggests that RNAs generated with RNAtranslator have a better energy landscape, which enhances their overall foldability and thermodynamic resilience. To provide a more comprehensive assessment of RNA stability, we incorporate the free energy unfolding of the ensemble ($\Delta G_{ensemble}$), which considers the entire thermodynamic ensemble of possible secondary conformations rather than just the lowest energy structure using ViennaRNA [68]. RNAtranslator generated RNAs have a distribution that tend to be slightly more negative than natural RNAs (Fig 7). The length of the RNA sequence also affects the secondary structure and binding affinity. It is also an important factor for diversity and novelty of the design. For this reason, we also compare the RNA length distributions across different groups. Again, as can be seen in Fig 7, RNAtranslator-generated RNAs span broader range than natural binding RNAs with higher median compared to natural non-binding RNAs. Stability analyses of RNAs generated for other five protein targets are presented in S6 Fig. Overall, compared to other methods, RNAtranslator generated RNAs display a more stable energy distribution with a large span of sequence lengths where RNAtranslator has a slight advantage. This reinforces RNAtranslator’s ability to adopt biologically relevant folding patterns under diverse conditions.

### RNAtranslator attends binding domains of the proteins more than other regions

**Fig 8:**
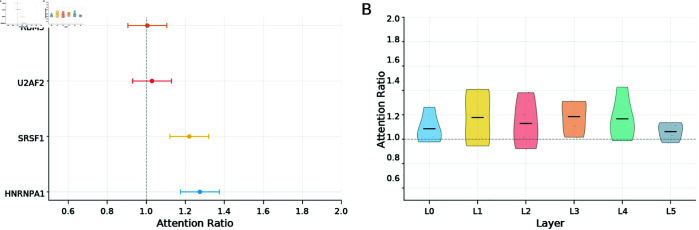
Analysis of RNAtranslator Attentions by visualizing the cross attentions This figure visualizes the cross-attention analysis of the decoder in RNAtranslator. The model is run on up to 1000 RNA–protein pairs per RNA-binding protein (RBP), collecting attention maps from all layers and heads. For each protein, the maximum attention weight received from any RNA position is attended. To assess the model’s focus on known RNA-binding domains, an attention ratio is defined as the maximum attention score within a known domain divided by the maximum score outside the domain. (A) Four representative RBPs exhibit attention ratios greater than one (dashed line), indicating that the model assigns higher attention to known binding regions. (B) When averaged across all RBPs, this attention ratio peaks in the middle decoder layers (L1–L4), suggesting these layers contribute most to identifying binding sites.

We analyze whether our model captures relevant binding features. To do so, we analyze cross-attention maps from RNA-protein interactions and compute residue-level attention scores. We compare attention to known functional domains and attention to other regions *attention ratio* (see Sect Methods). We show the distribution of attention ratios for 4 proteins in Fig 8A. It shows that for most proteins, the model is more strongly oriented toward known binding domains, with attention ratios larger than 1 (e.g., HNRNPA1, U2AF2, and SRSF1). This indicates that the model prioritizes functionally important regions. We also compare the attention ratio distributions of model layers in Fig 8B. We show that the attention ratio is larger than 1 for layers 1 to 4, indicating stronger focus on functional domains during these stages of decoding. In contrast, the first (0) and last (5) layers show lower and more variable attention ratios, implying that these layers may focus on general sequence features.

It is important to note that attention to regions outside of known functional domains does not indicate a failure of the model. RNA-protein binding is influenced not only by domain-specific recognition but also by structural features of the RNA, such as hairpins, bulges, and other secondary structure elements that contribute to RNA stability and binding context. The model may rightfully focus on such regions, which are not annotated as canonical binding domains but still play important roles in the interaction. In this analysis, we specifically examine attention to annotated protein domains to evaluate whether the model can recognize well-characterized binding signals. Also, to reduce noise and increase reliability, we aggregate results over multiple RNA-protein interaction samples per protein. This approach ensures that the observed enrichment reflects consistent model behavior rather than random fluctuations in individual samples.

## Discussion

We introduce a novel approach to protein-conditional RNA sequence design by formulating the problem as a natural language translation task. Unlike existing methods that rely on separate generation and optimization steps, our model, RNAtranslator, learns a unified latent space for RNA and protein interactions, enabling direct and target-specific RNA design. The results demonstrate that RNAtranslator outperforms state-of-the-art approaches in the generation of natural-like RNA sequences while improving binding affinity to the target. Our results suggest that the designed RNAs maintain stability while preserving sequence and length diversity. This balance is important, as excessive stability may lead to undesired rigidity which bars effective binding, while high flexibility may lack specificity in the design. Additionally, our docking and binding affinity evaluations confirm that RNAtranslator designs natural-like RNA sequences with superior binding potential compared to existing models.

The advantage and the novelty of RNAtranslator lies in its ability to generalize beyond predefined RNA-protein interactions. Traditional RNA design methods rely on either (1) experimental selection processes, which are costly and time-intensive, or (2) computational models that suffer from data scarcity and the need for post-generation optimization. We eliminate these bottlenecks by using large-scale RNA-protein interaction datasets and end-to-end training on paired data, allowing the model to learn implicit binding rules without manual intervention. Moreover, RNAtranslator does not rely on third-party binding affinity predictors, which are rarely available only for a few hundred well-studied proteins. For instance, we had to optimize the RNAs generated by RNAGEN using a binding affinity predictor trained specifically for RBM5 and we had to fine-tue the GenerRNA method with RNAs that are known to bind to RBM5. RNAtranslator does not require any of these steps as it was trained with large scale interaction data, end-to-end. This allows RNAtranslator to expand the scope of RNA-based therapeutics.

Despite these promising results, there are several areas for further improvement. One challenge is the scalability of training large language models on biological sequences, given the computational complexity of RNA-protein interactions. Future work could explore techniques such as parameter-efficient fine-tuning or self-supervised learning on larger, unlabeled datasets to further enhance performance. Another limitation is the lack of experimentally validated binding data for novel RNA designs. While our computational assessments suggest high-affinity interactions and molecular dynamics (MD) simulations offer insights into how molecules bind and how stable they are, they are not perfectly accurate. Also, errors may arise from initial conditions, boundary effects, and the inherent limitations of classical physics approximations that can introduce uncertainties. This is also the case for our binding score prediction model used in validation. DeepCLIP provides useful insights, but it also has some limitations. These models are trained on protein-RNA binding data from studies on specific proteins and, therefore, cannot be applied to predict binding scores of proteins for which no such data exists. In addition, some RNA-binding proteins (RBPs) interact with RNA sequence patterns and structures in a more unspecific manner, which makes the accurate prediction of binding sites more difficult. The performance of DeepCLIP also depends strongly on the quality of the training data. Some methodological biases in the CLIP datasets can result in models that recognize general CLIP sites rather than protein-specific sites. Experimental validation through techniques such as surface plasmon resonance or isothermal titration caliometry will be necessary to confirm binding efficacy in vitro [69].

In summary, our study presents a paradigm shift in protein-conditional RNA design by introducing an end-to-end generative approach that eliminates the need for post-optimization or fine-tuning. RNAtranslator successfully designs RNA sequences with enhanced stability, diversity, and binding affinity, overcoming key challenges in existing computational and experimental methods. Our findings suggest that this framework will play a pivotal role in the future of RNA therapeutics, molecular engineering, and synthetic biology.

## Methods

### Dataset

We collect RNA-Protein interaction data primarily from the RNAInter Database [70], which provides approximately 26 million interactions, combining both experimentally validated and computationally predicted RNA-protein interactions (RPIs). RNAInter is the largest and the most comprehensive RNA interaction dataset currently available. The dataset predominantly includes human interactions obtained from diverse high-throughput experimental methods, such as CLIP-seq, RIP-seq, and yeast three-hybrid assays, covering various RNA types like mRNA, lncRNA, miRNA, and snRNA. S1C Fig shows that mRNAs make up the majority of RNAs (44.9%), followed by lncRNAs (28.3%) and pseudogenes (18.8%). For proteins, most entries are generic proteins (95.2%), with a small portion classified as transcription factors (3.8%) and RNA-binding proteins (1.0%).

To ensure data quality and reduce errors associated with computational predictions in RNAInter, we use high-quality interaction data from CLIPdb and the Protein Data Bank (PDB) complexes for finetuning RNAtranslator. Specifically, we obtain human interaction data from CLIPdb using the POSTAR3 repository [71]. As shown in S1A Fig, we retrieve RNA and protein identifiers, and genomic coordinates from both RNAInter and POSTAR3 and then extract their sequences using publicly available APIs from UniProt [72], NCBI [73], Ensembl [74], and miRBase [75]. Then, we deduplicate RNAs associated with each protein using CD-HIT-EST [76] with a 90% identity threshold.

We observe a substantial imbalance between proteins in terms of their interaction counts in the CLIPdb dataset (see S1E Fig). The number of interactions range from as few as 85 interactions (for EZH2) to more than 1 million interactions (1,079,145 for ELAVL1). To avoid a potential bias, we use an up-sample the dataset to achieve equal representation. However, to prevent up-sampling from disproportionately amplifying specific RNA sequences, we cluster RNAs in the entire dataset using CD-HIT-EST with a 90% sequence identity threshold. Concurrently, we cluster proteins using CD-HIT at a 40% identity threshold, though this generates predominantly singleton clusters (one protein per cluster) due to the relatively low sequence similarity among proteins and then we apply a weighted sampling method where weights are inversely proportional to the size of each (protein, RNA) complex cluster. Here, we define a complex as a unique protein-RNA interaction pair, and complex clusters represent groups of interactions sharing the same protein cluster and RNA cluster assignments. For example, if 10 interactions belong to the same complex cluster, each interaction receives a weight of 0.1, while two interaction in a same cluster receives a weight of 0.5. Each unique complex cluster receives equal weight regardless of its original interaction count, effectively down-weighting over-represented RNA cluster combinations to prevent sequence redundancy and since protein clusters are small and mostly contain single proteins, they do not substantially alter the weighting scheme. This method prioritizes interactions involving less common RNA clusters, thereby preserving RNA sequence diversity and ensuring balanced protein representation by up-sampling. We also filter out sequences with extremely short or long lengths with minimum length of 10 and maximum length of 1,024 for both protein and RNA sequences. As shown in S1B Fig, this filtering step helps to eliminate outliers in the length distributions of protein and RNA sequences.

In addition, we further augment our fine-tuning dataset using the PRI30K [77] dataset, curated from protein-RNA complexes available in the Protein Data Bank (PDB). This dataset consists of protein-RNA complexes structured into pairwise interactions which contains approximately 30,000 unique interactions. We randomly sample a total of 12 million interactions to fine-tune our model from this dataset.

### Problem formulation

Let $p=(p_{1},\ldots ,p_{n_{p}})$ denote a protein sequence, where each *p*_*i*_ is a token representing one or multiple amino acids chosen from the standard set of 20 amino acids. Let $r=(r_{1},\ldots ,r_{n_{r}})$ be the corresponding RNA sequence we aim to generate, where each *r*_*i*_ is composed exclusively of the nucleotides {A, C, G, U}. Our primary objective is to learn the conditional distribution p(r∣p), which describes how likely an RNA sequence *r* is given a protein sequence *p*. By formulating this as a language translation problem—where the protein sequence is treated as the source language and the RNA sequence as the target language—we use techniques from natural language processing (NLP) to model conditional protein-RNA design.

We factorize p(r∣p) via the chain rule:

$$p(r|p)\;=\;\prod_{i=1}^{n_{r}}p(r_{i}|r_{<i},p)$$ (1)

We then train a model with parameters *θ* that minimizes the negative log-likelihood (NLL) over a protein-RNA interaction dataset $𝒟=\{(p^{\left(1\right)},r^{\left(1\right)}),\ldots ,(p^{\left(|𝒟|\right)},r^{\left(|𝒟|\right)})\}$:

$$NLL(𝒟)\;=\;-\sum_{k=1}^{\left|𝒟\right|}\sum_{i=1}^{n_{r}^{\left(k\right)}}\log\;p_{\theta }(r_{i}^{\left(k\right)}|r_{<i}^{\left(k\right)},\;p^{\left(k\right)}).$$ (2)

### Model architecture

We implement the RNAtranslator using the T5 architecture [78], a widely adopted encoder-decoder transformer designed for sequence-to-sequence tasks.

#### Training.

We feed the encoder with the protein sequence *p* to model the protein language and learn the information from a protein’s RNA-binding domains (RBDs). Before processing, each amino acid token is converted into a learned token embedding, using BPE tokenization to handle large protein vocabularies. Positional information is also encoded, following the T5 architecture [79], which uses learned relative positional encoding instead of fixed absolute positions. Unlike sinusoidal or learned absolute encodings, T5 introduces a relative position bias directly into the self-attention mechanism. Each attention score between a query and a key token is adjusted by a bias term that depends only on their relative distance. This allows the model to focus on the distance between tokens rather than their exact positions, making it more flexible to handle sequences of variable length and capture meaningful patterns. A tokenized protein sequence of length *n*_*p*_ is mapped to a sequence of embeddings, which is then passed through layers of self-attention and feedforward blocks. The final output of the encoder is a matrix $H\in {\mathbb{R}}^{n_{p}\times d}$ where *d* is the dimension of the model and encodes contextual information about the amino acid composition of the protein, potential RNA-binding domains and other functionally relevant features. Given the output of the encoder *H*, the decoder generates the target RNA sequence $\left(r_{1},\ldots ,r_{n_{r}}\right)$ one token at a time. At each timestep *i*, the decoder conditions on, (i) the partially generated RNA sequence $\left(r_{1},\ldots ,r_{i-1}\right)$ via *self-attention*, (ii) the encoder output *H* via *cross-attention*:

$$D_{i}={Dec}_{\theta }((r_{1},\ldots ,r_{i-1}),H)$$ (3)

Here, *D*_*i*_ denotes the hidden representation of the decoder at timestep *i*. This vector $D_{i}\in {\mathbb{R}}^{d}$ captures contextual information from both the evolving RNA sequence and the features of the encoded protein. Finally, a linear layer $W_{\text{vocab}}\in {\mathbb{R}}^{d\times |𝒱|}$ projects *D*_*i*_ onto the vocabulary space 𝒱 of RNA tokens, and a softmax function normalizes these scores to yield a probability distribution over all possible next tokens.

#### Inference.

At inference time, we produce a *de novo* RNA sequence $\hat{r}$ for a given protein *p* by sampling tokens sequentially from the output distribution:

$${\hat{r}}_{1}\sim p_{\theta }(r_{1}|p),\;{\hat{r}}_{2}\sim p_{\theta }(r_{2}|{\hat{r}}_{1},p),\;\ldots \;{\hat{r}}_{n_{r}}\sim p_{\theta }(r_{n_{r}}|{\hat{r}}_{<n_{r}},p).$$ (4)

There are various techniques for sampling in language models [80–82]. For our work, we consider multiple approaches. One approach is greedy sampling, which selects the token with the highest probability at each time step. While this method often produces coherent sequences with high overall likelihood, it can be overly conservative and may lead to repetitive outputs. To mitigate this, we also use beam search. Beam search [80] maintains multiple candidate sequences by exploring the top *B* tokens (where *B* denotes the beam size) at each decoding step, eventually selecting the sequence with the highest overall probability. Although beam search generally improves sequence quality, it may still limit diversity. To introduce greater diversity, we incorporate stochastic sampling techniques such as top-*k* sampling and nucleus sampling (also known as top-*p* sampling). In top-*k* sampling [81], the token selection is restricted to the *k* most probable tokens, thereby reducing the likelihood of sampling low-probability tokens that could reduce sequence quality. Nucleus sampling, on the other hand, dynamically determines the candidate set based on a cumulative probability threshold *p*, ensuring that only the tokens contributing to the majority of the probability mass are considered. Both stochastic methods provide a balance between randomness and determinism, enabling the generation of RNA sequences that are diverse yet biologically plausible.

Recognizing that no single sampling strategy perfectly balances diversity and quality of the generated RNAs, we generate a pool of candidate RNA sequences using various sampling strategies and hyperparameter settings. We then apply scoring using predicted binding score, consistency, and minimum free energy (MFE). All of these metrics are normalized between zero and one, to score the generated sequences effectively. We normalize the MFE using min-max scaling $MFE_{norm}=1-\frac{MFE-\min(MFE_{gen})}{\max(MFE_{gen})-\min(MFE_{gen})}$ by this we ensure that lower MFE values are ranked higher. Binding score is derived from DeepCLIP model [37] and consistency measures the frequency of a given RNA sequence appearing across different sampling runs, normalized by dividing by the maximum occurrence observed $C_{norm}=\frac{C}{\max(C_{gen})}$. Higher consistency values indicate more robust sequence generation across sampling strategies.

S1D Fig shows a comparative analysis of different sampling strategies using RNA pools generated for nine selected proteins. As shown in the figure, we evaluate several sampling strategies by adjusting hyper-parameters, such as the top-k value and temperature, to determine the optimal combination that maximizes RNA quality scores. Among these sampling strategies, the top-k sampling method with *k* = 30 and the temperature set to 1.5 consistently achieves the highest score. Given its superior performance, we employ this sampling strategy throughout all RNA generation and evaluation processes described in this study.

### Experimental setup.

We trained RNAtranslator on two NVIDIA TITAN RTX GPUs using Fully Sharded Data Parallel (FSDP) to split the training across devices efficiently. we tokenize RNA and protein sequences using the Byte Pair Encoding (BPE) algorithm. A vocabulary size of 1,000 tokens was chosen to balance representation quality and computational efficiency. Each sequence is truncated or padded to a fixed length of 1,024 tokens to ensure uniformity during model training. We set a batch size of 8 and used gradient accumulation over 16 steps. We also applied weight decay with the Adam optimizer. The training carried on for 350,000 iterations which took just over a month. Inference takes a couple of seconds on the same device. Our hardware configuration used FP16 with the optimization level O1 and a fixed seed of 42 for reproducibility. The model has a stack of 6 identical encoder-decoder layers with 12 attention heads. The total number of parameters is 41.4 million. In fine-tuning, we used three NVIDIA L40S GPUs, and increased the batch size to 15, conducting this phase for an additional 75,000 iterations.

We trained GenerRNA on the same computational setup used for RNAtranslator. Specifically, we obtained a base GenerRNA model and fine-tuned it using RNA interactions from the CLIPdb database, employing 3,917 interactions for RBM5 and over 1 million interactions for ELAVL1. We fine-tuned for 50,000 iterations. For RNAGEN, we used the released pre-trained model and generated 500 sequences which are optimized to bind to RBM5 and ELAVL1 using the DeepBind model [14] for the corresponding protein.

### Evaluation metrics

Our primary objective is to ensure that the RNAs generated by RNAtranslator closely resemble natural RNAs in both structural properties and binding characteristics. To achieve this, we first define a set of properties that capture stability (e.g., minimum free energy, GC content) and binding specificity (e.g., affinity scores). We then compare the *distributions* of these properties with natural RNA sequences. To ensure a fair and comprehensive evaluation, we validate the generated RNAs by comparing their distribution. We use the DeepCLIP model [37] to quantify the RNA-protein binding affinity given a protein and RNA sequence. This model predicts the probability of binding based on features that correlate with known protein-binding motifs and RNA sequence patterns. For each target protein, we divide the RNA interaction dataset from CLIPdb into training, validation, and test sets with proportions of 80%, 10%, and 10%, respectively. We then train a DeepCLIP model using the partitioned data and evaluate its performance on the test set to confirm its reliability for further evaluations. The results of this evaluation are presented in S3 Fig, which indicate that the modes are effective in estimating binding affinity. For validation, we specifically select 500 RNAs from the test set as representatives of natural binding RNAs.

Furthermore, beyond sequence-based validation, we also analyze the designed RNAs at the structural level and simulate RNA-protein complexes using docking and moleculdar dynamics simulations. Since ground-truth structure for the designed RNAs are not available, we predict the structure using RhoFold+. Using HDOCKlite, we model the RNA-protein complex. HDOCKlite is a lightweight version of the HDOCK docking algorithm that focuses on RNA-protein interactions, incorporating a scoring function optimized for molecular docking [52]. Through these docking and MD analyses, we evaluated the interactions of the generated RNA sequences with their target proteins, assessing docking scores, potential complex structures, and the stability and dynamic properties of each RNA-protein interaction.

### Molecular dynamics simulations

We use molecular dynamics simulation framework OpenMM [41] to assess structural dynamics of the designed RNAs. Simulations employ an isothermal-isobaric ensemble (NPT) at 310 K and 1.0 atm using a Langevin integrator. Each system undergoes energy minimization, followed by equilibration and a production run utilizing CUDA-accelerated computing. Systems with initial potential energies exceeding 10^6^*kJ*/*mol* triggered extended minimization protocols with up to 10,000 total steps. Trajectories are recorded at regular intervals to track conformational changes over time. This standardized protocol is applied to all systems. We simulated the three disease related proteins, mentioned in section Results in a solvated environment, specifically system is solvated in a TIP3P water box with 1.0*nm* padding and neutralized at 0.15 M ionic strength by adding appropriate numbers of *Na*^ + ^ and *Cl*^−^- ions. Before running simulations, we preprocess the structures with pdbfixer [53] to correct missing or nonstandard residues and remove unnecessary heterogens, then prepare using the AMBER14 force field, ensuring appropriate protonation states at pH 7.0.

To quantify binding affinity during simulations, we calculate the interaction energy (ΔE) using:

$$\Delta E=E_{\text{complex}}-(E_{\text{protein}}+E_{\text{RNA}})$$ (5)

where $E_{\text{complex}}$, $E_{\text{protein}}$, and $E_{\text{RNA}}$ represent energies of the RNA-protein complex, isolated protein, and isolated RNA, respectively. A more negative ΔE signifies stronger and more favorable interactions between the protein and RNA [83]. We further evaluate several structural and dynamic metrics to gain deeper insights into binding characteristics. Specifically, we quantify the number and stability of hydrogen bonds (H-bonds), as a higher number of stable H-bonds typically correlates with stronger binding interactions [84–86]. Hydrogen bonds are identified using a donor–acceptor distance cutoff of 4.5 and a donor–hydrogen–acceptor angle cutoff of 150^°^, following commonly accepted geometric criteria. Additionally, the number of atomic contacts is calculated between the two molecular components, where any pair of atoms within a distance of 0.45*nm* (4.5) is counted as a contact. A greater number of such close contacts generally corresponds to a larger and potentially more stable binding interface [86,87].

We also computed the free energy landscape (FEL) based on molecular dynamics simulations by selecting a group of atoms in the protein and using the first frame of the simulation as a reference. For each subsequent frame, we calculate the root-mean-square deviation (RMSD) which measures how much the atomic positions differ from the reference and the radius of gyration (RG) which reflects the spread of the atoms around their center of mass. These two values are then used to construct a two-dimensional histogram that estimates the probability density of observing particular RMSD and RG combinations. To smooth out any noise, we apply a Gaussian filter to the histogram. The free energy landscape is calculated using the relation

F=−kTln(P), (6)

where *kT* represents the thermal energy (with *k* as the Boltzmann constant and *T* as the temperature) and *P* is the probability density from the smoothed histogram. By shifting the minimum free energy value to zero, we can clearly determine the depth of the landscape, which indicates the energy barrier between the most stable and less stable states. A larger depth indicates a higher energy barrier, meaning the complex is more stable [88,89].

### Attention map analysis

In attention map analysis, we focus specifically on the cross-attention layers of the decoder. We use the attention patterns to analyze whether the model focuses on functionally meaningful regions in the protein sequence. We randomly select up to 1,000 RNA-protein interaction samples for each protein of interest. For each pair, we tokenize the protein and RNA sequences and then feed them into a trained RNAtranslator model. During the forward pass, we extract the decoder’s cross-attention maps, which represent how each pair of RNA-protein positions are attended. These maps are collected from every decoder layer and attention head. For each head, we compute a summary score by taking the maximum attention weight across all RNA positions for each protein token. These head-level scores are then averaged to obtain layer-level attention profiles. Finally, we average the attention scores across all layers to get a global protein-level attention profile per interaction.

Since RNAtranslator operates on BPE tokens, we decode each protein token back into amino acid residues. We then distribute the attention score of each token equally across its constituent amino acids. This gives us an attention score for every residue in the protein, allowing a direct comparison with known functional regions. To assess whether the model focuses on biologically meaningful sites, we compare attention scores in known functional domains of the protein (e.g., RNA recognition motifs, PIWI domains) with scores outside these regions. We define the *attention ratio* as the maximum attention in binding domains divided by the maximum attention outside domains. A higher ratio indicates stronger attention within functionally important regions.

## Supporting information

S1 Text Noise robustness and length analyses. (DOCX)

S1 Fig Overview and processing of the RNA-protein interaction dataset. (A) Interaction data is collected from RNAInter and CLIPdb. RNA and protein identifiers, sequences, and genomic positions are retrieved using external APIs such as UniProt and NCBI. (B) Filtering is applied to remove extremely short or long sequences, reducing outliers in the RNA and protein length distributions. The effect of filtering is illustrated by the shift between the “All Data” and “Filtered Data” distributions. (C) Using the dataset annotations, RNA and protein families are extracted to analyze their distribution. As shown in the Figure, the majority of RNAs in the dataset are mRNAs (44.9%), followed by long non-coding RNAs (lncRNAs) and pseudogenes. On the protein side, more than 95% are general proteins, with smaller fractions classified as transcription factors or RNA-binding proteins. (D) Several sampling strategies for RNA selection are evaluated, varying top-*k* values and temperature parameters. The configuration with top-*k* = 30 and temperature  = 1.5 yields the highest RNA quality scores. (E) The dataset exhibits a strong imbalance in the number of interacting RNAs per protein, with some proteins associated with very few interactions and others with over a million (Left). To address this, oversampling is employed to ensure a balanced and diverse set of protein–RNA pairs (Right).
(TIFF)

S2 Fig Comparison of RNA secondary structures targeting therapeutic proteins. Secondary structure visualizations of RNAs binding to three target proteins: EGFR (A), p53 (B), and thrombin (C). In each panel, the top row displays the structure of a validated RNA, while the bottom row shows an RNA sequence generated by the RNAtranslator model. Each structure is annotated with its minimum free energy (MFE) and normalized minimum free energy (N-MFE). The generated RNAs often display folding patterns with multiple loops and stable helices, suggesting potential for effective protein binding.
(TIFF)

S3 Fig DeepCLIP performance on the test data. DeepCLIP models are trained for individual RNA-binding proteins using the training dataset and evaluated on the corresponding test set. The figure presents receiver operating characteristic (ROC curves and area under the curve (AUC) values for nine proteins: ZC3H7B, AGO2, U2AF2, RBM5, HNRHNPA1, ELAVL1, TARDBP, MOV10, and SRSF1. Each curve plots the true positive rate against the false positive rate, with the dashed line indicating the performance of a random classifier. The models demonstrate high predictive accuracy for most proteins, achieving AUC values of 0.95 for ZC3H7B and ELAVL1, 0.92 for TARDBP, and 0.96 for MOV10. Some proteins, including U2AF2 (0.77) and HNRHNPA1 (0.78), show lower AUC values, which suggests weaker but still meaningful predictive performance.
(TIFF)

S4 Fig Robustness to input noise and sequence length. (A) Evaluation of RNAtranslator’s robustness to noise in the input protein sequence for ELAVL1 and RBM5. Random mutations are introduced at increasing rates, and binding scores remain high up to a 25% mutation rate, after which performance declines sharply. This indicates that the model is resilient to moderate levels of input noise. (B) This Figure shows the assessment of the model’s performance across varying RNA sequence lengths. RNAtranslator generates high-scoring binders across a broad range of RNA lengths, with stable performance observed for both RBM5 and ELAVL1.
(TIFF)

S5 Fig Distribution of predicted binding scores for PRPF8. The binding scores are predicted using the DeepCLIP model, and the distribution of these scores shows that RNAtranslator sequences for PRPF8 behave similarly to natural binding RNAs. In contrast, natural non-binding RNAs have much lower scores and are clearly separated.
(TIFF)

S6 FigStability analysis comparing RNAtranslator-generated and natural RNAs.RNAtranslator-generated RNAs show similar Minimum Free Energy (MFE) and Ensemble Free Energy (ΔG ensemble) distributions to natural binding RNAs, indicating thermodynamic and structural stability. GC content analysis confirms structural robustness, and sequence length distribution highlights their broader range.(TIF)
