# Supplementary material for: RNAtranslator: Modeling protein-conditional RNA design as sequence-to-sequence natural language translation
Source: PLoS Comput Biol. 2025 Oct 3;21(10):e1013541. doi: 10.1371/journal.pcbi.1013541 (PMC12510665; doi:10.1371/journal.pcbi.1013541)
Supplement: S1 Text — (DOCX) [file pcbi.1013541.s001.docx]

**S1 Text. Noise Robustness and Length Analyses.**

We perform a robustness analysis to understand how our model behaves under variations in the input protein sequence and changes in the target RNA length. First, we test the model’s sensitivity to noise by introducing random mutations in the protein sequences. Specifically, we randomly replace amino acids to simulate noise levels ranging from 0% to 60%. After generating RNAs based on these noisy inputs, we calculate the average binding scores. As shown in S4A Fig, the model maintains high binding performance up to around 25% noise. Beyond this level, the scores drop sharply, indicating reduced reliability. This shows that the model is robust to small sequence changes, but larger distortions can significantly affect performance.

Next, we evaluate how well the model performs when generating RNAs of different lengths. We design sequences across a wide range, from very short RNAs like siRNAs and miRNAs (18–25 nt), to medium-length RNAs (51–100 nt), and up to long non-coding RNAs (401–600 nt). Intermediate types include short regulatory RNAs (26–50 nt), long RNAs (101–200 nt), and shorter long non-coding RNAs (201–400 nt). S4B Fig shows that the model consistently produces high-scoring RNA binders across all these length ranges. While there are small variations depending on the protein target, the overall binding performance remains stable, demonstrating that our model can generalize well to different RNA length requirements.
