# Supplementary figures and images for: RNAtranslator: Modeling protein-conditional RNA design as sequence-to-sequence natural language translation
Source: PLoS Comput Biol. 2025 Oct 3;21(10):e1013541. doi: 10.1371/journal.pcbi.1013541 (PMC12510665; doi:10.1371/journal.pcbi.1013541)

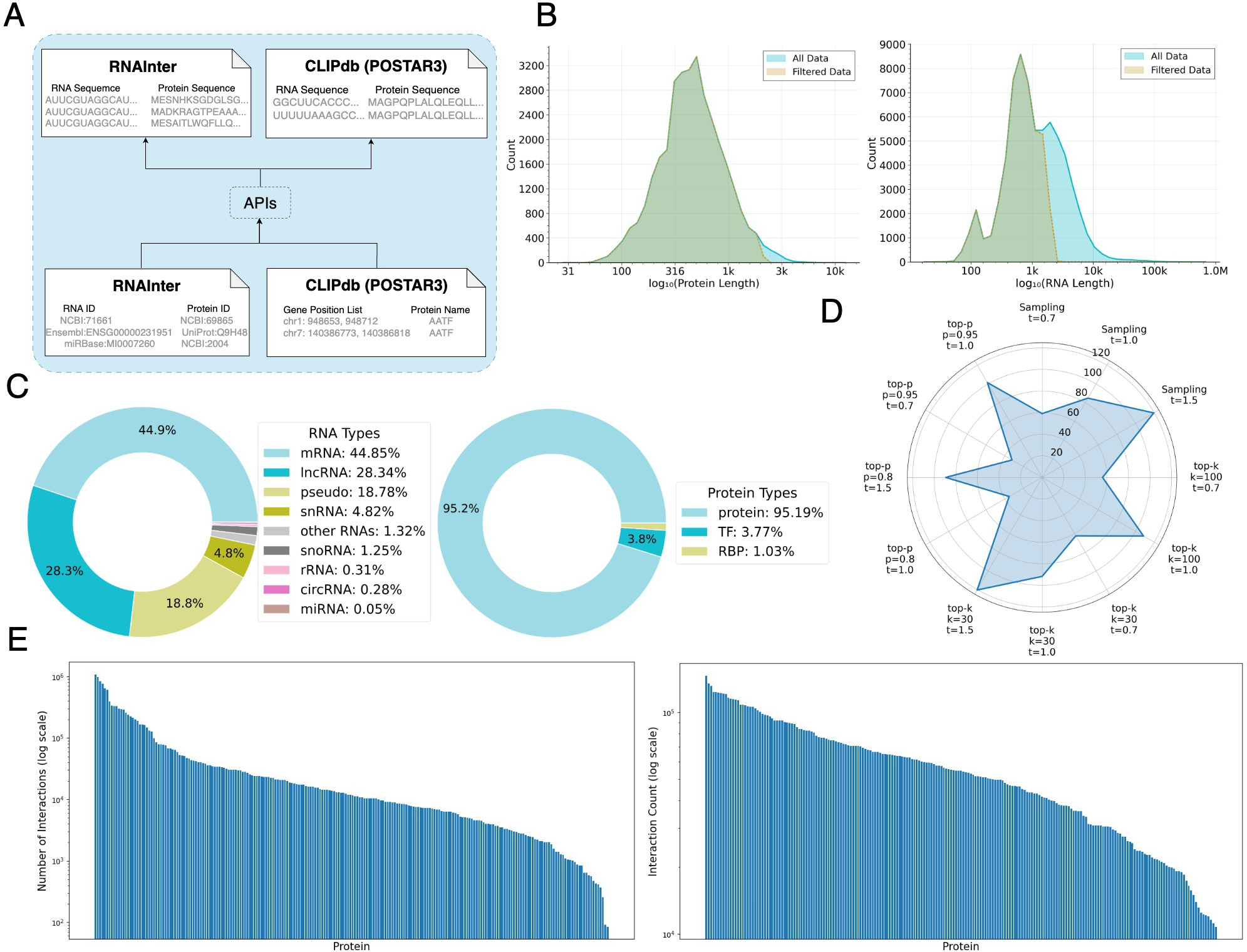

Supplement: S1 Fig — (A) Interaction data is collected from RNAInter and CLIPdb. RNA and protein identifiers, sequences, and genomic positions are retrieved using external APIs such as UniProt and NCBI. (B) Filtering is applied to remove extremely short or long sequences, reducing outliers in the RNA and protein length distributions. The effect of filtering is illustrated by the shift between the “All Data” and “Filtered Data” distributions. (C) Using the dataset annotations, RNA and protein families are extracted to analyze their distribution. As shown in the Figure, the majority of RNAs in the dataset are mRNAs (44.9%), followed by long non-coding RNAs (lncRNAs) and pseudogenes. On the protein side, more than 95% are general proteins, with smaller fractions classified as transcription factors or RNA-binding proteins. (D) Several sampling strategies for RNA selection are evaluated, varying top-k values and temperature parameters. The configuration with top-k = 30 and temperature = 1.5 yields the highest RNA quality scores. (E) The dataset exhibits a strong imbalance in the number of interacting RNAs per protein, with some proteins associated with very few interactions and others with over a million (Left). To address this, oversampling is employed to ensure a balanced and diverse set of protein–RNA pairs (Right). (TIFF) [file pcbi.1013541.s002.tif]

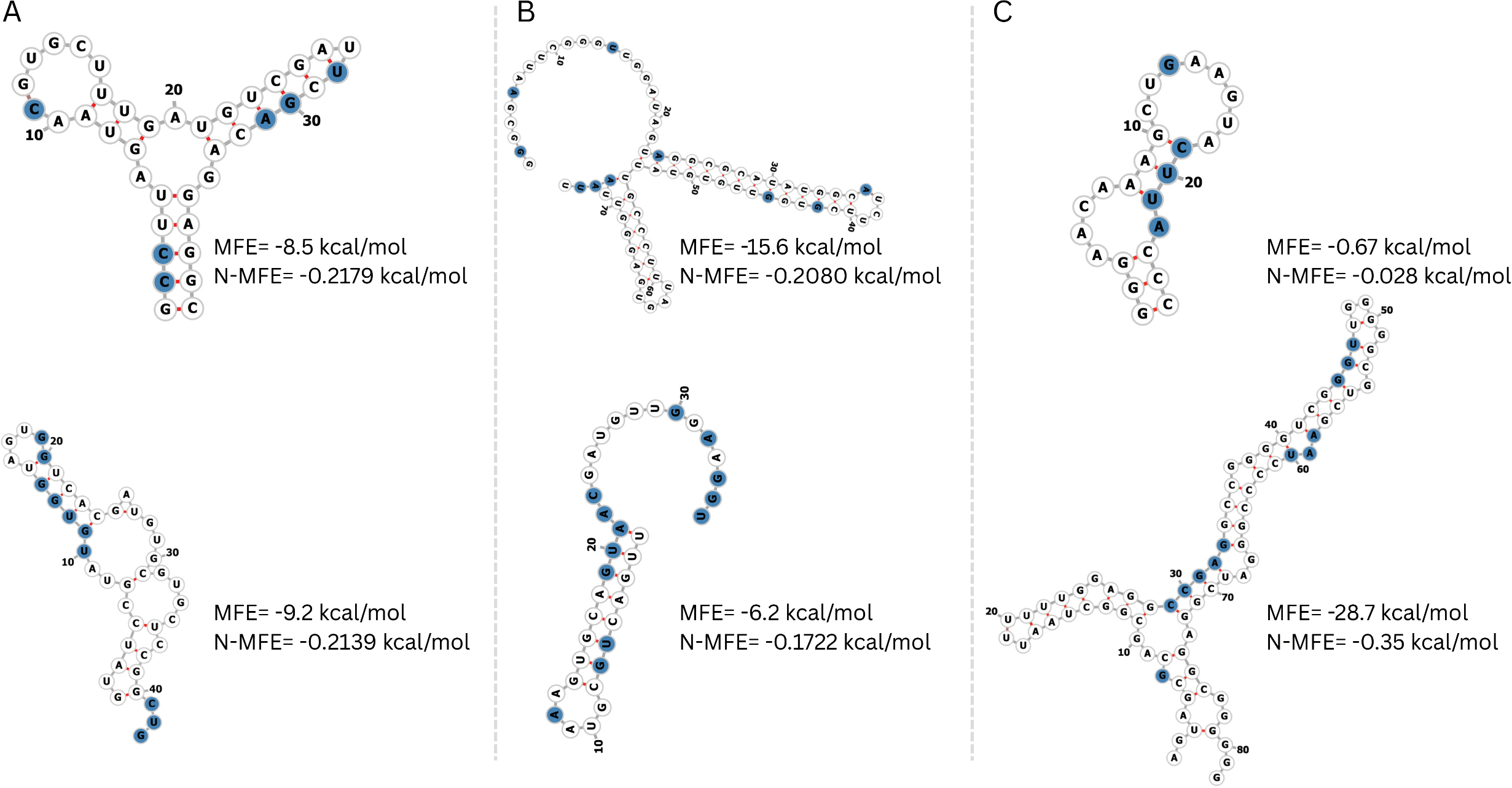

Supplement: S2 Fig — Secondary structure visualizations of RNAs binding to three target proteins: EGFR (A), p53 (B), and thrombin (C). In each panel, the top row displays the structure of a validated RNA, while the bottom row shows an RNA sequence generated by the RNAtranslator model. Each structure is annotated with its minimum free energy (MFE) and normalized minimum free energy (N-MFE). The generated RNAs often display folding patterns with multiple loops and stable helices, suggesting potential for effective protein binding. (TIFF) [file pcbi.1013541.s003.tif]

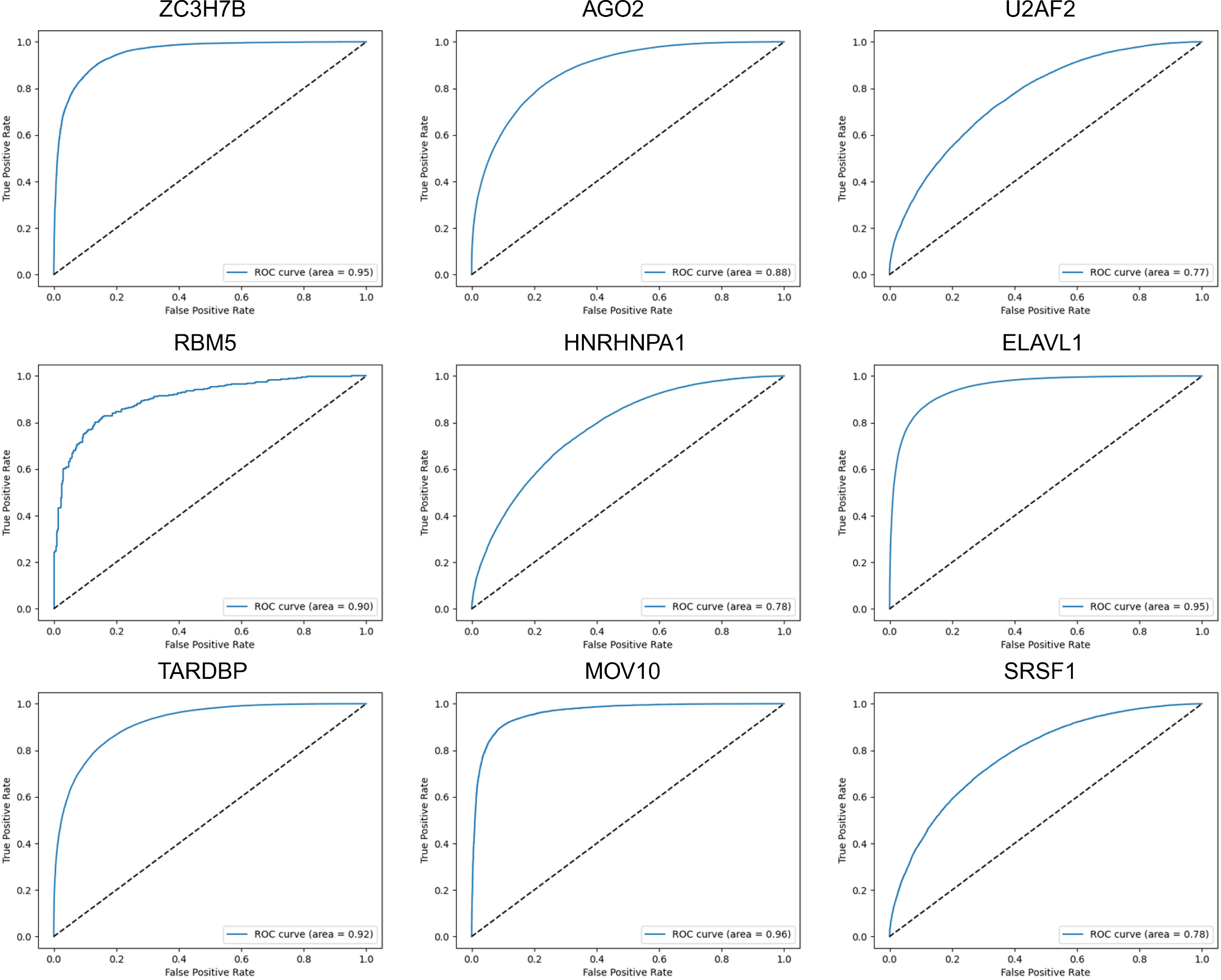

Supplement: S3 Fig — DeepCLIP models are trained for individual RNA-binding proteins using the training dataset and evaluated on the corresponding test set. The figure presents receiver operating characteristic (ROC curves and area under the curve (AUC) values for nine proteins: ZC3H7B, AGO2, U2AF2, RBM5, HNRHNPA1, ELAVL1, TARDBP, MOV10, and SRSF1. Each curve plots the true positive rate against the false positive rate, with the dashed line indicating the performance of a random classifier. The models demonstrate high predictive accuracy for most proteins, achieving AUC values of 0.95 for ZC3H7B and ELAVL1, 0.92 for TARDBP, and 0.96 for MOV10. Some proteins, including U2AF2 (0.77) and HNRHNPA1 (0.78), show lower AUC values, which suggests weaker but still meaningful predictive performance. (TIFF) [file pcbi.1013541.s004.tif]

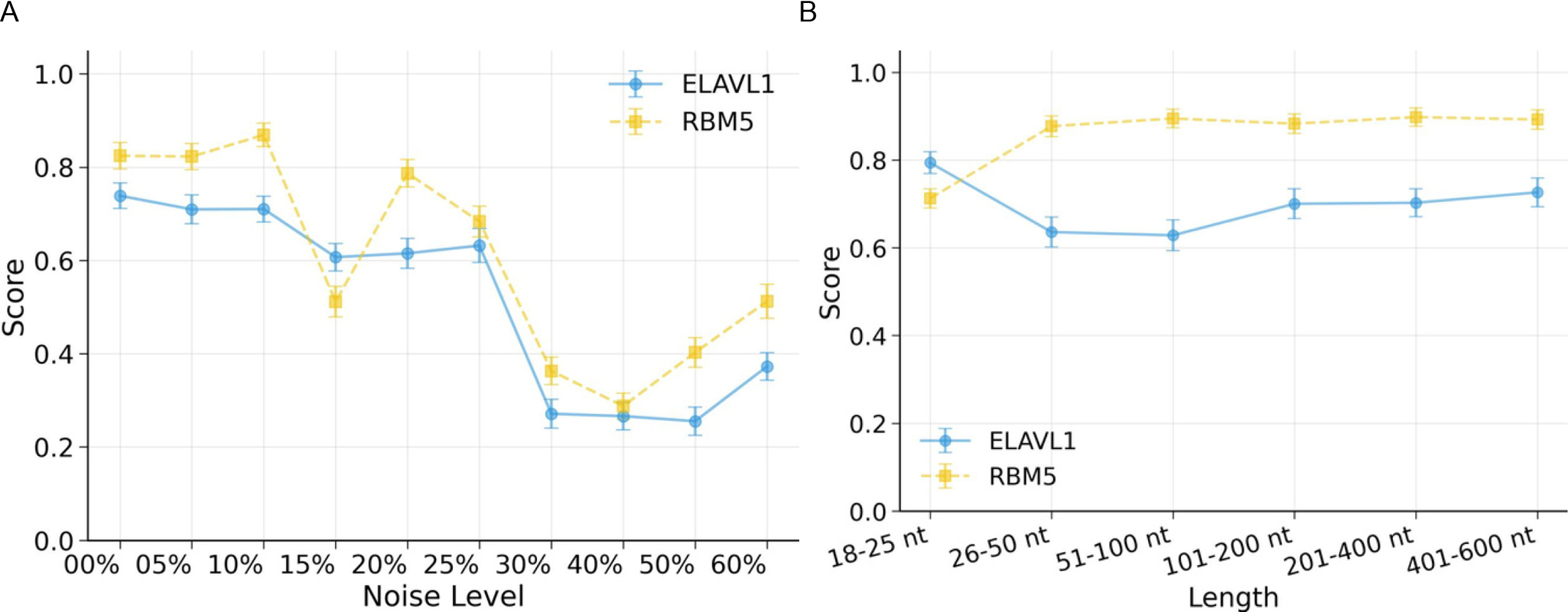

Supplement: S4 Fig — (A) Evaluation of RNAtranslator’s robustness to noise in the input protein sequence for ELAVL1 and RBM5. Random mutations are introduced at increasing rates, and binding scores remain high up to a 25% mutation rate, after which performance declines sharply. This indicates that the model is resilient to moderate levels of input noise. (B) This Figure shows the assessment of the model’s performance across varying RNA sequence lengths. RNAtranslator generates high-scoring binders across a broad range of RNA lengths, with stable performance observed for both RBM5 and ELAVL1. (TIFF) [file pcbi.1013541.s005.tif]

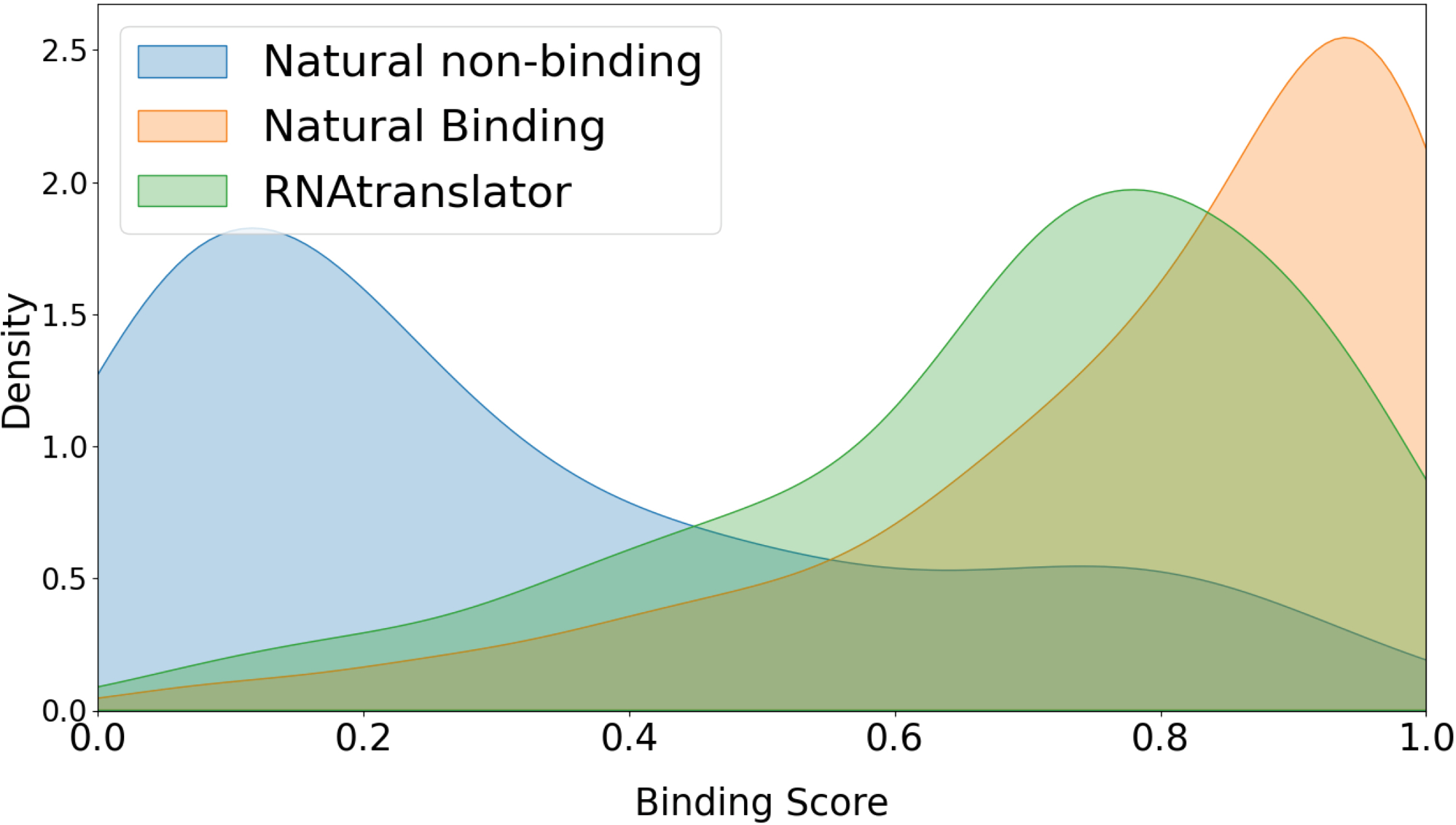

Supplement: S5 Fig — The binding scores are predicted using the DeepCLIP model, and the distribution of these scores shows that RNAtranslator sequences for PRPF8 behave similarly to natural binding RNAs. In contrast, natural non-binding RNAs have much lower scores and are clearly separated. (TIFF) [file pcbi.1013541.s006.tif]

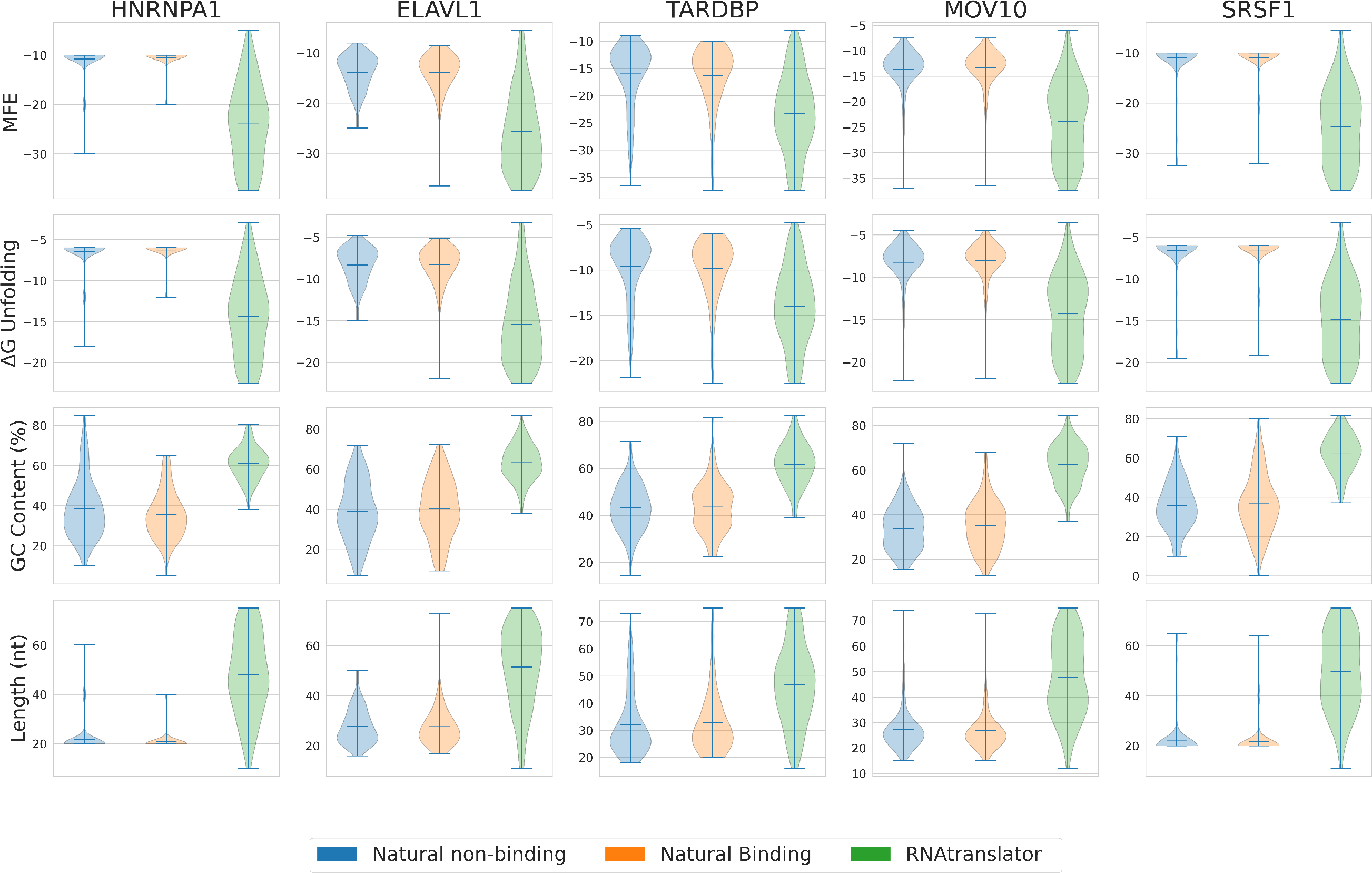

Supplement: S6 Fig — RNAtranslator-generated RNAs show similar Minimum Free Energy (MFE) and Ensemble Free Energy (ΔG ensemble) distributions to natural binding RNAs, indicating thermodynamic and structural stability. GC content analysis confirms structural robustness, and sequence length distribution highlights their broader range. (TIF) [file pcbi.1013541.s007.tif]
